# Determination of Seroprevalence of Borrelia burgdorferi IgG in Adult Population Living in Trabzon

**DOI:** 10.4274/balkanmedj.2015.0478

**Published:** 2017-01-05

**Authors:** Merve Cora, Neşe Kaklıkkaya, Murat Topbaş, Gamze Çan, Asuman Yavuzyılmaz, İlknur Tosun, Faruk Aydın

**Affiliations:** 1 Department of Medical Microbiology, Karadeniz Technical University School of Medicine, Trabzon, Turkey; 2 Department of Public Health, Karadeniz Technical University School of Medicine, Trabzon, Turkey; 3 Trabzon Provincial Health Directorate, Trabzon, Turkey

**Keywords:** Lyme borreliosis, seroprevalence, tick, ELISA, western blot

## Abstract

**Background:**

Lyme borreliosis is a tick-borne, multi-systemic infectious disease that is thought to be wide spread in Turkey even though studies on its seroprevalence are limited.

**Aims:**

To determine the seroprevalence of Lyme borreliosis in part of north-eastern Tur-key (in the city of Trabzon), and to identify possible relationships between seropositivity and various factors such as location, gender, age group, occupation, income, and educational level.

**Study Design:**

Retrospective cross-sectional study.

**Methods:**

A total of 884 blood samples collected from provincial and district health centers serving a population of about 800,000 were included in this study. ELISA was used to determine the anti-Borrelia IgG antibody levels in the samples. Samples that yielded positive results by ELISA were further subjected to western blot (WB).

**Results:**

IgG antibodies were found in 128 samples (14.5%). Statistical analysis revealed significant differences between age groups and educational levels in terms of the incidence of seropositivity, whereas location, gender, occupational group and income level had no effect (p<0.001, p<0.001, p=0.948, p=0.645, p=0.131, p=0.080 respectively).

**Conclusions:**

The risk of contracting Lyme borreliosis in Trabzon is high, and necessary measures need to be taken to avoid the spread of disease.

Lyme borreliosis (LB) is a tick-borne disease caused by a group of pathogenic spirochetes belonging to B. burgdorferi sensu lato complex or Lyme borrelia and is transmitted by Ixodes spp ticks (1). Within this group of bacteria B. burgdorferi sensu stricto, B. garinii, and B. afzelii are known to be responsible for causing LB in humans. The most common agent that causes LB in the United States is B. burgdorferi sensu stricto while in Asia LB is commonly caused by B. garinii and B. afzelii. All three species are reported to be common in Europe. In recent years, it has been reported that other species of Borrelia such as B. spielmanii, B. valaisiana, and B. lusitania are also associated with LB (2).

Lyme borreliosis is a multisystemic zoonosis that can be divided into three clinical phases. Although the early localized phase is characterized by erythema migrans (EM), non-specific flu-like symptoms may also be seen at this stage (3). In addition to two or more EM, neurological, rheumatic, and cardiac manifestations may also occur in the early disseminated phase. The late phase emerges with arthritis or acrodermatitis chronica atrophicans (ACA), and neurological symptoms may occur, but only rarely (4,5).

Lyme borreliosis can be clinically diagnosed if the classical signs and symptoms are present. However, the same signs and symptoms cannot be observed in all patients at every stage. In addition, the disease may exhibit similarities to different diseases in different clinical situations (1,3). For this reason, various laboratory tests are needed to diagnose LB. In order to determine the etiological agent, microscopy, culture, and polymerase chain reaction (PCR) can be used. However, these methods are most sensitive in the detection of B. burgdorferi from the EM lessions, that is not a common laboratory test, and also time-consuming, labor-intensive, as well as it is not sensitive enough (1,3). For these reason serological tests are important in diagnosis. The Centers for Disease Control and Prevention (CDC) proposed a two-step approach for serological testing in order to identify active disease or past infections. An ELISA or immunofluorescent antibody test (IFA) is recommended as the first-line test, after which western blot (WB) is required as a second-line confirmatory test (6,7,8). The IgM and/or IgG antibodies occur in only 20–50% of patients in the early localized stage. In the early disseminated phase, the seropositivity of IgM and/or IgG is 70–90%. IgG antibodies are detectable in all patients in the late phase (9).

Lyme borreliosis is the most common tick-borne disease in North America, Europe, parts of Asia, and northern Japan (4). There are a limited number of studies evaluating B. burgdorferi antibody positivity in Turkey. The majority of studies were carried out on patients with arthritis, rheumatism and similar complaints, uveitis, Behcet’s disease, morphea, lichen sclerosis, facial paralysis, aseptic meningitis or manifestations similar to those of LB using ELISA, and B. burgdorferi antibodies were found at different levels of prevalence (0-66.6%) in these groups. Other studies performed using ELISA included only individuals considered to be in the at-risk group due to living in a village or dealing with livestock farming, and in these the positivity rate ranged from 3.28 to 35.9% (9). Ixodes spp. ticks are frequently seen in the Eastern Black Sea Region including the city of Trabzon. The prevalence of Ixodes spp. infected with B. burgdorferi sensu lato was reported as 15.9% in the Black Sea Region (10). A case study was carried out in 1990 concerning the presence of LB in the city of Trabzon (9). The study included 90 serum samples obtained from people including those involved in livestock farming. The samples were analyzed by ELISA, and the proportion with anti-Borrelia IgG antibodies was 6.6% (11). However, there are no recent data on the incidence of LB in the north-eastern part of Turkey. The aim of the study was to determine the seroprevalence of B. burgdorferi sensu lato infection among healthy people from Trabzon province and its districts and the relation between anti-Borrelia IgG antibodies and selected socio-demographic factors.

## MATERIALS AND METHODS

### Study population

The serum samples used in this study were collected from 884 healthy adult individuals between the ages of 20 and 79 living in Trabzon city center and nine counties during the period from August 2007 to August 2008 and were stored at -80 ºC until analysis performed in 2012. The mean age of the study group was 40.8 years, and the female/male distribution was 51.24% and 48.76%, respectively. This study was approved by the local Ethics Committee of Karadeniz Technical University School of Medicine. Individuals whose serum samples were included in this study were informed about the aim of the study, and they gave verbal consent. The sample size required was calculated as at least 884 with 50% expected prevalence, 95% confidence level, and 3% deviation using the formula (where n is sample size, is z-score, p is estimated proportion, and d is desired precision). Nine of 17 counties in the province of Trabzon were selected for sampling based on the geographical features of the provinces (12).

### Serological tests

A commercial IgG ELISA kit (Immunolab GmbH; Kassel, Germany) was used to determine the B. burgdorferi s.l. IgG antibodies. According to the manufacturer, the Immunolab B. burgdorferi IgG ELISA test kit contains a whole cell antigen extract of B. burgdorferi sensu stricto, which cross-reacts with B. afzelii and B. garinii, plus pure OspC, which increases the specificity and sensitivity of the assay. In the western blot (WB), a commercial B. burgdorferi IgG (Euroimmun; Lubeck, Germany) kit was used to evaluate the samples that had been found to be positive by ELISA. These kits were prepared using complete antigens of B. afzelii, and a recombinant VlsE antigen. Tests were conducted in accordance with the manufacturer’s instructions, and WB results were evaluated using the EUROLINEScan (Euroimmun; Lubeck, Germany) program. Only the serum samples that gave positive results for both ELISA and WB were accepted as positive and included in the statistical analysis.

### Socio-demographic data

In order to determine the factors affecting the anti-Borrelia antibodies, the results were compared in terms of socio-demographic data (e.g. location, gender, age, educational level, occupational group, and income level). The participants were classified as high-risk and low-risk groups in terms of occupa-tion (Table 1). Farmers, skilled and unskilled workers, police officers, soldiers, housewives, and retirees were considered as being in the high-risk group, whereas teachers, health personnel, office workers, directors, secretaries, craftsmen, engi neers, clergy, drivers, media, accountants, students, and unemployed people were classified in the low-risk group.

### Statistical analysis

Chi-square tests were conducted using Statistical Package for the Social Sciences 13.0 software (SPSS Inc.; Chicago, IL, USA), and differences were considered significant at p<0.05.

## RESULTS

A total of 236 (26.7%) out of 884 examined serum samples were found to be seropositive for anti-Borrelia IgG antibodies by ELISA. Of these, 128 (14.5%) were also positive in the WB (Table 2). To evaluate the positivity rates according to location, residential areas were classified as being in the city center or the rest of the city. According to the western blot, anti-Borrelia IgG antibodies were present in 78 (14.4%) out of 541 samples from the city center, and in 50 (14.6%) of 343 samples obtained from individuals living in other districts (Table 3). The difference in incidence of Lyme seropositivity between the city center and the districts (14.4%, 14.6%; respectively) was not significant (p=0.948). However, there was a significant relationship between B. burgdorferi IgG positivity and age group and the educational level of patients (p<0.001, p<0.001; respectively) (Table 2). B. burgdorferi IgG positivity increased as educational level decreased, and was more common in those without formal education. Examination of the positivity results in terms of gender, occupation, and income level did not yield any signifi cant difference between groups (p=0.645, p=0.131, p=0.080 respectively) (Table 2).

## DISCUSSION

A total of 884 serum samples obtained from individuals living in Trabzon city center and districts was screened by ELISA first, and then positive samples were further examined by WB in order to determine the seropositivity of B. burgdorferi. A total of 26.7% of examined individuals were positive according to the ELISA results while 128 of those (14.5%, overall) were also found to be positive according to the WB results. These findings led to the conclusion that when working with the ELISA method only, a high rate of false positivity may be obtained, and therefore the results should be confirmed with further WB tests as recommended by the CDC (6). The number of previous studies specific to the north-eastern part of Turkey is limited and included fewer samples than in the current study. One study reported a seropositivity of 6.6% according to ELISA of 60 samples only (11). The relation between positive results and investigated parameters was quite limited. A review of the literature identified two publications from the eastern Black Sea region about LB (11,13). One presented a case of LB in 1990 (13). In the other, 60 individuals engaged in animal husbandry (30 of them settled in mountainous areas, the other 30 people settled in coastal areas), and 30 healthy individuals not engaged in animal husbandry living in Trabzon were sampled, with a 6.6% seropositivity rate according to ELISA in both groups (11).

In another study carried out along the north-western coast of Turkey (Düzce province) the seroprevalence of IgG among 349 forest workers and farmers was 10.9%, while 2.6% of 193 healthy controls showed seropositivity. The difference between the two groups was statistically significant (14). A wide range of seroprevalence levels for B. burgdorferi IgG antibodies was reported in similar studies conducted in Turkey, such as 2.0% in Erzurum (15), 17.6% in northern Cyprus (16), 35.9% in Antalya (17), 3.3% in Samsun (18), 0.0% in Sivas, 6% in Ankara, 7.8% in Izmir, 18.9% in Denizli, and 17% in Isparta (9).

According to the National Notifiable Disease Surveillance System’s data collected from 1992 to 2006 in the USA, the number of reported cases increased by 101% over this period (9908 cases in 1992, 19 931 cases in 2006). Looking at the average ratio of the incidence of LB in states during this period, it was determined that the ratio changed from 0.01 to 73.6/100,000 persons. The lowest incidence was 0.01 in Colorado and Montana, while it was 73.6 in the state of Connecticut (4). Numerous studies have been conducted on the seroprevalence of LB in Europe. The seroprevalence of B. burgdorferi IgG was reported to be 54% in Austria in 2006 (19), 2.27% in Northern Italy in 2010 (20), 25% in Southern Poland in 2009 (21), 9.4% in Germany (22), 9.6% in Western Norway (23), and 9.6% in Northern Spain (24). In a study carried out in eight provinces in China, the seroprevalence of B. burgdorferi IgG was 3-15% (25). The incidence of LB in southern Sweden was 69/100 000 in 1995 (26), whilst the rate of seroprevalence of Borrelia IgG antibodies was 3.2% among young children in 2010 (27).

In some studies, seropositivity was assessed according to the socio-demographic characteristics of individuals as reported in the current study. The distribution of the rates of seropositivity according to place of residence was analyzed in Germany, revealing higher rates in rural areas (22). Similarly, the seroprevalence was higher outside the city center in this study. Similarly, the seroprevalence was higher outside the city center in this study.

When the IgG seroprevalence was examined according to sex, it was more common in men in the USA (4), Austria (19), Southern Poland (21), Germany (22), Western Norway (23), and China (25), whereas in Northern Spain (24) it was more common among women; meanwhile the incidence was almost the same in men and women in South Sweden (26). In studies conducted in Turkey, it was reported as being more common among men in Düzce (14), and more common among women in Antalya (17) and Samsun (18). In this study, the seropositivity was comparable in women (15.0%) and men (13.9%) with no significant difference between the two.

In studies that evaluated the IgG seroprevalence with regard to age, a bimodal distribution was found in the USA (mostly in ages 5-9 and 55-59) (4) and South Sweden (mostly in ages 5-9 and 60-74) (26). In other studies, the highest rates were found between the ages 60-69 in Austria (18), ≥50 in Southern Poland (21), 70-79 in Germany (22), 60-69 in Western Norway (23), 11-20 in Northern Spain (24), and 40-49 in China (25). In Turkey, on the other hand, the highest rates were found between the ages 10-20 in Düzce (14), 20-39 in Antalya (17), and 15-39 in Samsun (18). The highest rate of seropositivity was found in those aged ≥70 in the current study (32.7%). This result was attributed to the fact that retired indi-viduals spend more time in rural areas.

In terms of the relationship between seropositivity and profession, it was found that seropositivity was more common among site workers than among office workers in Southern Poland (21), while no difference in seroprevalence was found in terms of occupation in Northern Spain (24). In our study, seropositivity was more common in the high-risk group of occupations (15.9%) than in the low-risk group of occupations (12.2%), which is consistent with the literature.

Analysis of the data according to income level showed no significant difference in seropositivity between groups (15.7% in the group earning ≤1000 TRY, 11.0% in the group earning >1000 TRY; p=0.080). In contrast there was a significant correlation between level of education and seropositivity: 27.0% in those without a formal education, 15.8% in those who graduated from primary school, and 10.4% in those who graduated from high school and higher (p<0.001).

The occurrence of negative results in WB from some of the samples that gave positive results in the ELISA may be due to cross reaction of Lyme borrelia with other pathogens. The 14.5% seropositivity rate obtained in this study indicates that there is a high risk of getting LB in Trabzon. The lack of significant difference in the positivity rates between the city center and districts and between males and females could be due to the high mobility of the city’s residents. The increase in seropositivity with age could be due to older individuals spending more time in nature and/or rural areas than younger individuals. The lower seropositivity in those with a higher educational level could be attributed to their higher awareness and the taking of necessary precautions when performing outdoor activities.

The different seropositivity rates in the different regions of Turkey indicate that environmental factors such as climate and living conditions might affect the seropositivity of LB, and this needs further investigation. The climate is suitable for both ticks and the reservoir animals found in Turkey, which could increase the seropositivity of LB in Turkey. These findings indicate that the disease should be kept in mind as a differential diagnosis for patients with Lyme-like symptoms.

## Figures and Tables

**Table 1:**
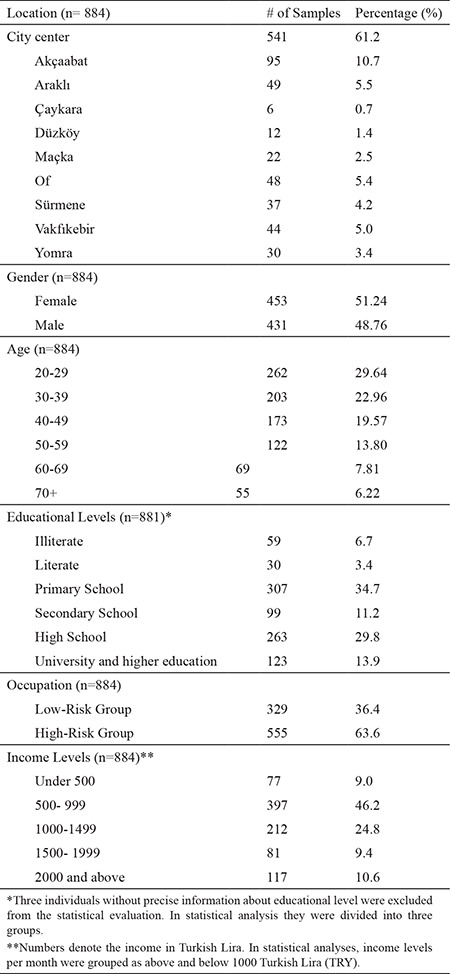
Grouping the samples according to socio-demographic characteristics

**Table 2:**
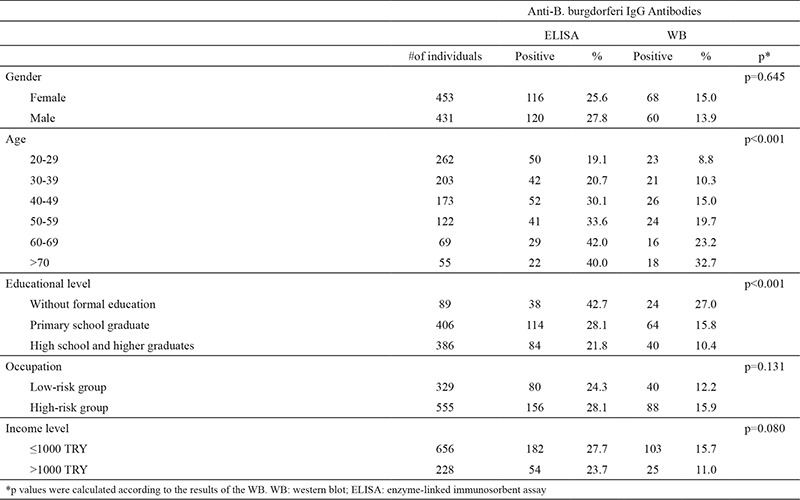
Distribution of seropositivity according to socio-demographic characteristics of samples

**Table 3:**
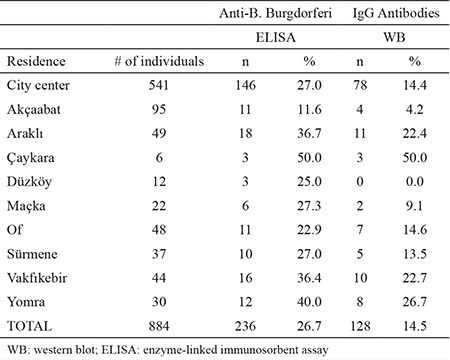
Number of positive results and corresponding percentages according to area of residence
